# Prospective Randomized Comparison of Opioid-Based Versus Non-Opioid-Based Anaesthetic Protocols for Endobronchial Ultrasound-Guided Transbronchial Needle Aspiration (EBUS-TBNA)

**DOI:** 10.3390/jcm14061964

**Published:** 2025-03-14

**Authors:** Anna Szelka-Urbanczyk, Maja Copik, Hanna Misiolek, Ewa Olewnicka, Maria Mirek, Szymon Białka

**Affiliations:** Department of Anesthesiology and Intensive Care, School of Medicine with Division of Dentistry in Zabrze, Medical University of Silesia, 41-800 Zabrze, Poland; ania.szelka@gmail.com (A.S.-U.); hanna.misiolek@gmail.com (H.M.); s84143@365.sum.edu.pl (E.O.); s84200@365.sum.edu.pl (M.M.); szymon.bialka@gmail.com (S.B.)

**Keywords:** sedation techniques, patient comfort in bronchoscopy, local anesthesia in EBUS, analgesia in endobronchial procedures, safety in bronchoscopic procedures, anesthetic protocols, endobronchial ultrasound, transbronchial needle aspiration, transtracheal block

## Abstract

**Background:** The evolution of interventional pulmonology has necessitated the refinement of anesthetic techniques to ensure the safety and efficacy of procedures such as endobronchial ultrasound-guided transbronchial needle aspiration (EBUS-TBNA), particularly when performed outside the conventional operating room setting. The management of anesthesia in patients with significant comorbidities, classified as American Society of Anesthesiologists (ASA) class III, presents distinct challenges. In this context, the transtracheal block emerges as a viable alternative to total intravenous anesthesia (TIVA) for these high-risk procedures. Objectives: This study aims to evaluate the comparative safety and efficacy of opioid-based intravenous analgesia versus a regimen combining sedative agents with transtracheal block in the administration of anesthesia for EBUS TBNA in high-risk patients. Design: We conducted a randomized observational study involving 57 elective EBUS-TBNA patients classified as ASA class III. **Methods:** Participants were allocated into two cohorts: one receiving intravenous opioid analgesia and the other receiving a combination of sedative agents with transtracheal block. Collected data encompassed patient demographics, medical history, incidence of adverse events during anesthesia, indicators of sympathetic nervous system activation, patient satisfaction levels, and the procedural conditions as assessed by the operator. **Results:** Opioid anesthesia was associated with increased desaturation (95.7% vs. 60.6%; *p* < 0.05) and higher pain-related sympathetic responses (VAS and SCI at 40–100; *p* < 0.05). No differences in other adverse events, patient satisfaction, or procedural conditions were noted. **Conclusions:** In summary, the study indicates that transtracheal block combined with sedatives may be a safer anesthetic alternative to opioid-based regimens for high-risk EBUS-TBNA patients, reducing desaturation and pain-related sympathetic activity without affecting satisfaction or procedural efficacy.

## 1. Introduction

Endoscopic techniques are increasingly preferred in medicine, offering less invasive alternatives to traditional surgery and blurring the lines between surgical and non-surgical disciplines. Interventional pulmonology, rapidly advancing, provides methods for quick and precise diagnostics and interventions, once exclusive to thoracic surgery. These include endobronchial stenting and cryotherapy, expanding treatment to high-risk patients. Anesthesia for these procedures varies from local to general, depending on the intervention’s complexity and patient needs [1].

Endoscopic procedures often occur outside the operating theatre, requiring a fully equipped anesthesia workstation and post-procedure observation with SpO_2_ and ECG monitoring. Guidelines suggest anesthesiologists manage sedation for high-risk patients [2].

Endobronchial ultrasound-guided transbronchial needle aspiration (EBUS-TBNA) is a minimally invasive method for diagnosing lung and mediastinal conditions, using ultrasound to guide biopsy collection [3]. This technique is safe, efficient, and has a low complication rate [4,5,6]. Nevertheless, some serious complications can arise, including pneumothorax, hemothorax, bleeding, or infection [7]. These complications are rare, but possible. The prevalence in the literature is estimated to be around 10% for bleeding and less than 1% for pneumothorax [8,9].

Patients undergoing these procedures may experience discomfort, which can complicate the process and affect sample quality. Anesthesia choice should consider patient health and facility capabilities, with some centers performing EBUS TBNA without an anesthesiology team unless necessary. The current challenge is to develop an anesthesia model for EBUS TBNA that is safe for high-risk patients and allows for precise biopsy collection [10,11]. Comparing common anesthesia approaches with opioid-free techniques could optimize protocols, especially for patients with significant comorbidities [12]. Pain assessment is essential in such comparison, especially for non-communicative patients, such as those anesthetized [13]. The Skin Conductance Index (SCI) is an objective method unaffected by external factors that measures autonomic responses to pain stimuli [14,15,16,17,18].

A transtracheal block is a type of local block where an anesthetic is injected directly in front of the trachea to block the nerve supply to the larynx and trachea [19]. This can be used as part of an opioid-free anesthesia approach during EBUS-TBNA procedures to minimize pain and discomfort without compromising the airway. The advantage of this technique is targeted pain relief while maintaining protective reflexes and avoiding the respiratory depression associated with systemic opioids [20].

The study aims to compare opioid-based anesthesia with opioid-free anesthesia in EBUS TBNA procedures, evaluating adverse events, patient satisfaction, procedural conditions, and sympathetic nervous system activation to determine the best approach for patient outcomes and procedural efficiency.

## 2. Methods

### 2.1. Trial Design and Participants

The study was structured as a prospective, randomized trial with ethical approval from the Silesian Medical University’s Bioethics Committee (Decision No. KNW/022/KB1/40/16). It was conducted at the Clinic of Anesthesiology and Intensive Care, Zabrze, Poland, from December 2016 (start of the recruitment phase) to March 2019, following CONSORT guidelines. Inclusion criteria were adults aged 18–75, with ASA III status, scheduled for elective EBUS TBNA, with no contraindications to the study’s drugs or methods, who gave informed consent. Exclusion criteria included absence of consent, contraindications to transtracheal block or study drugs, inability to use the Pain Monitor (Med.-Storm Innovations; Oslo; Norway) device, known drug allergies, or existing tracheotomy.

Baseline assessments included age, gender, height, weight, blood pressure, heart rate, oxygen saturation, respiratory rate, and comorbidities.

### 2.2. Randomisation, Blinding, and Sample Size

Patients were randomized using computer generated numbers (generated by random.org) accessed on 1 December 2016 to either Group I (24 patients) or Group II (33 patients). The study was not blinded.

### 2.3. Interventions

All patients received an IV infusion of saline with magnesium sulfate 10 min before endoscopy and oxygen at 5 L per minute. Group I was sedated with remifentanil at 0.05 μg/kg/min and propofol at 1 mg/kg. Group II received a transtracheal block with 5 mL of 2% lidocaine after a 0.5 mg/kg propofol dose, followed by a 1 mg/kg propofol bolus before endoscopy. Additional propofol at 0.25 mg/kg was administered as needed.

Hemodynamic stability was managed with ephedrine for MAP below 60 mm Hg or systolic pressure drops of 20% or more, and atropine for heart rates below 45 bpm. Airway patency issues were addressed with oropharyngeal tubes, and hypoventilation was managed with assisted ventilation.

Monitoring included continuous ECG, blood pressure every five minutes, pulse oximetry, and skin conductance for stress response. Adverse events and procedural conditions were documented, with the latter rated by the operator on a 1–5 Likert scale. Patient satisfaction with anesthesia was also evaluated using a five-point Likert scale approximately 6 h after emergence of anesthesia (the questions asked can be found in Supplementary Materials).

The Pain Monitor device by Med.-Storm Innovations, using Skin Conductance Index (SCI), was used to assess sympathetic nervous system activity during the procedure. It offers a reliable pain assessment, with 90% sensitivity and 74% specificity, by measuring skin conductance to evaluate pain intensity and the Awaking Index to monitor sedation depth, aiding in preventing unintended patient arousal during anesthesia. This objective and patient-centered tool is particularly valuable for non-communicative patients. Pain Monitor readings were segmented into fifteen-second intervals. Within these intervals, skin conductance variability (expressed as the number of peaks per second) was measured. The frequency of each value during the procedures was recorded and interpreted using the manufacturer’s Pain Monitor Index scale. This scale was used to correlate the skin conductance variability with the Visual Analog Scale (VAS) scores, with specific peak frequencies corresponding to different levels of perceived pain:

- 0.00–0.07 peaks/second indicating no pain or a VAS score of less than 40.
- 0.27 peaks/second correlating with a VAS score between 40 and 50.
- 0.33 peaks/second corresponding to a VAS score of 60–80.
- 0.4–0.7 peaks/second indicating a VAS score of 80–100.

### 2.4. Statistical Methods

The data were processed as follows for statistical analysis:

Nominal (dichotomous) scale variables were analyzed by tabulating the frequency of each outcome. For measurable variables on an interval or ratio scale, positional parameters such as the mean and standard deviation were calculated. The distribution’s normality was assessed using the Shapiro–Wilk test.

For nominal scale variables, the chi-square test of independence was employed, applying Yates’ correction for continuity when necessary. If the expected frequencies were too low for the chi-square test to be reliable, Fisher’s exact test was used instead.

For measurable variables, where significant deviations from a normal distribution were detected by the Shapiro–Wilk test, the nonparametric Mann–Whitney U test was utilized to compare the two groups.

The data were then contextualized with the duration of each individual procedure. The results of the statistical analysis were organized and presented in both tabular and graphical formats to facilitate interpretation.

Statistical significance was determined using predefined thresholds, with a *p*-value greater than 0.05 indicating non-significance and a *p*-value less than 0.05 indicating statistical significance. All statistical analyses were performed using Microsoft Excel (part of the MS Office suite) and Statistica software version 13.0 (TIBCO Inc., Palo Alto, CA, USA), with the analyses conducted in the Polish language edition of the software.

## 3. Results

**Figure 1:**
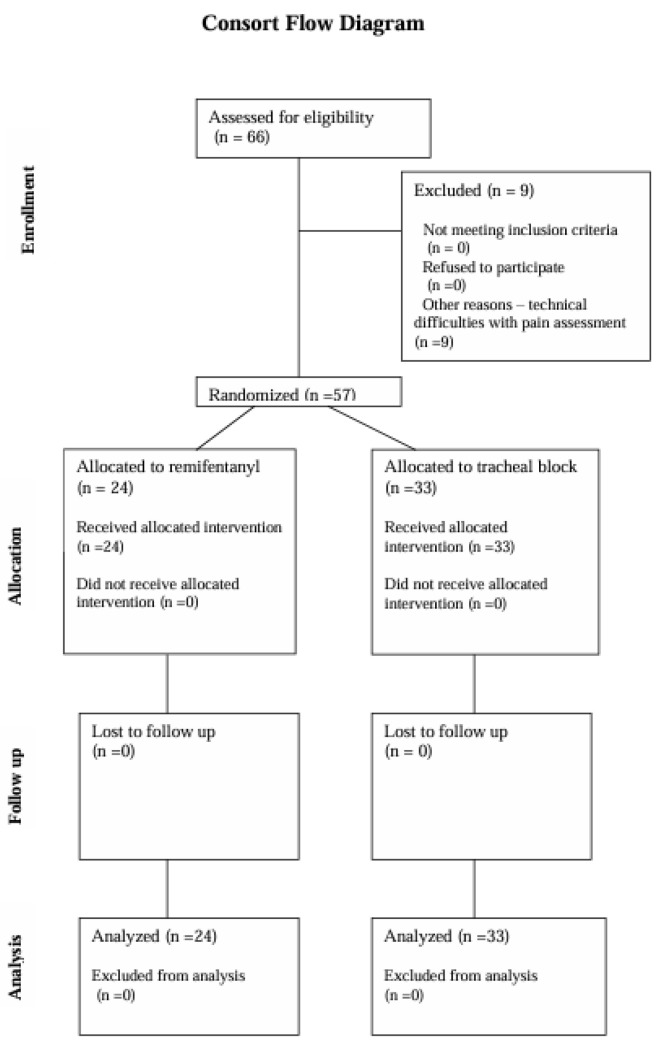
CONSORT flow diagram.

Patient Demographics and Study Participation: The study initially included 66 patients who provided written, informed consent to participate and were scheduled for transbronchial biopsy of mediastinal lymph nodes. Of these, 57 patients completed the study. Nine patients were excluded due to technical issues such as electrode detachment and monitor circuit disconnections, which introduced artifacts that precluded the assessment of the Pain Monitor recordings (Figure 1).

Group Characteristics: Upon comparing demographic data, no statistically significant differences were found between the two groups (Table 1). Similarly, the prevalence of comorbidities did not differ significantly between the groups (details available in Supplementary Materials).

**Figure 2:**
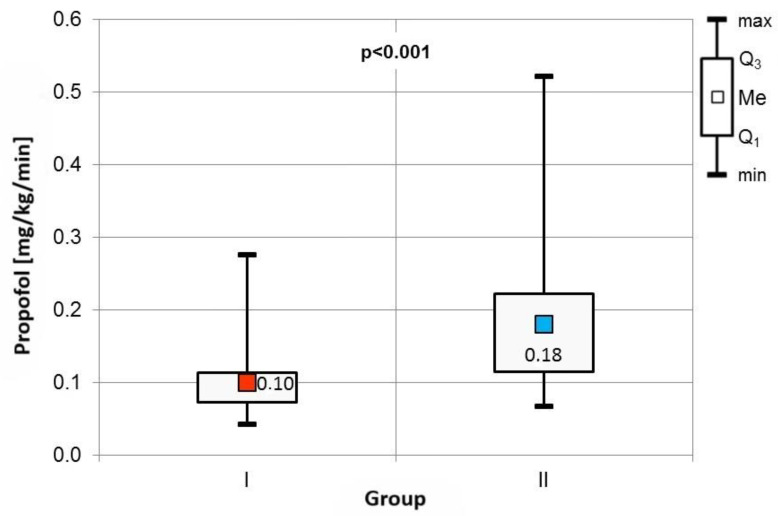
Comparison of propofol consumption between the groups.

The frequency of specific indications for Endobronchial Ultrasound-Guided Transbronchial Needle Aspiration (EBUS TBNA) was comparable across both cohorts (Table 2). Procedure durations, as well as baseline blood pressure, heart rate, and percutaneous blood saturation levels, showed no significant differences between the groups. However, Group II exhibited a significantly higher consumption of propofol, adjusted for patient body weight and procedure duration (Figure 2).

Adverse Events and Procedure Assessment: Adverse events were carefully monitored in both groups. Group I experienced a significantly higher incidence of desaturation events (Table 3). No differences were observed in the occurrence of bradycardia or apnea, and hypotension was not reported in any participant. Patient satisfaction with anesthesia and operator assessment of procedural conditions did not differ significantly between the groups.

**Figure 3:**
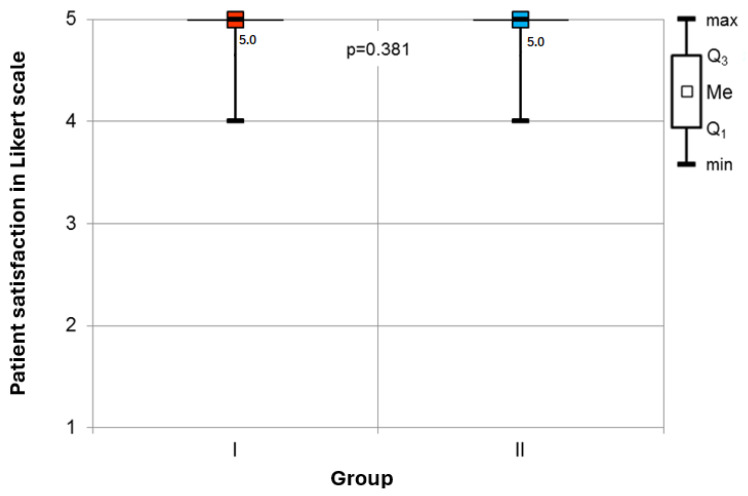
Comparison of patient-reported satisfaction rates in the groups.

Patient Satisfaction: The analysis revealed no statistically significant difference in patient-reported satisfaction with anesthesia among the surveyed cohort. Patients in both groups were satisfied with anesthetic management (Figure 3).

**Figure 4:**
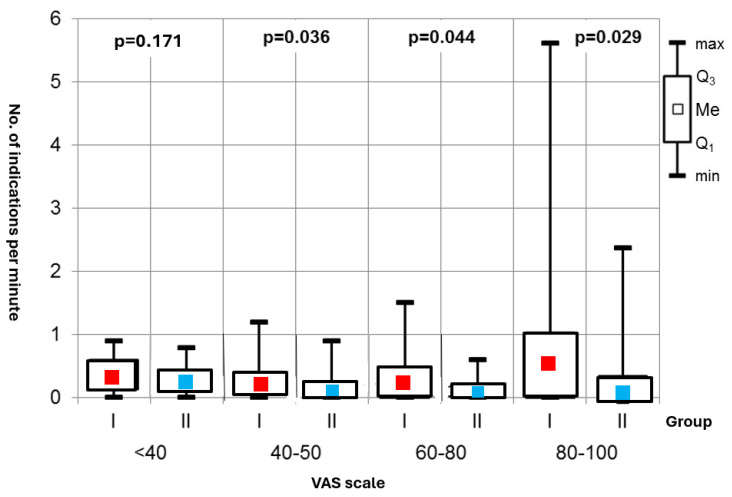
Number of indications per minute.

Sympathetic Activity: A key finding was the statistically significant difference in episodes of increased sympathetic activity during the procedures between the two groups—Group I showed a notably higher frequency of strong sympathetic responses, which were associated with pain experiences on the Visual Analog Scale (VAS) at intensity levels of 40–50, 60–80, and 80–100 (Figure 4).

**Figure 5:**
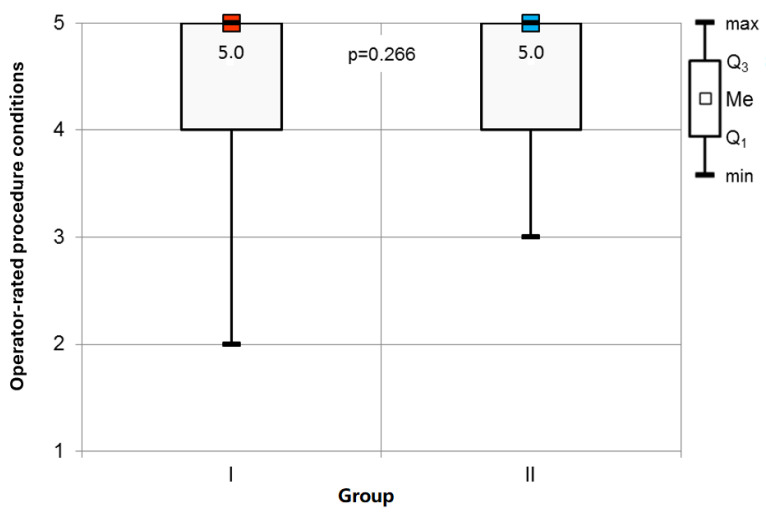
Operator-rated procedure conditions assessment.

Study Conditions: Statistical analysis indicated no significant variance in the operators’ assessments of the procedural conditions among the respondents (Figure 5).

## 4. Discussion

Our research has shown that a customized anesthesia approach for EBUS TBNA in ASA III patients can greatly improve safety and procedural outcomes. We found that using a transtracheal block instead of systemic opioids could shift interventional pulmonology towards local anesthesia, minimizing respiratory issues. Our protocol combines propofol’s sedative qualities with magnesium sulfate’s bronchodilation benefits, avoiding benzodiazepines to reduce respiratory depression risk. This approach is particularly effective for older patients with multiple health conditions [21].

Comparative studies have shown the transtracheal block to be superior to other local anesthesia techniques, such as the “spray-as-you-go” method, due to its consistent anesthetic effect [22]. This preference is likely due to the effective dispersion of the anesthetic agent during coughing, which enhances the block’s efficacy. The transtracheal block ensures a more uniform and extensive distribution of lidocaine, leading to improved local anesthesia and potentially reducing the need for systemic sedatives [23,24].

Patient satisfaction with anesthesia was high across both studied groups, which reflects a contrast with other oncological diagnostic procedures like bronchofiberoscopy or tumor biopsies that are typically performed under local anesthesia and may not provide the same level of comfort. The “pleasant, blissful sleep” induced by propofol was noted by many participants, despite occasional pain at the injection site [25,26].

The operators also rated the conditions for conducting the examination favorably across both groups. In centers without an anesthesiology team, the operator independently manages sedation, drug selection, dosage, and therapeutic decisions based on patient monitoring. Focusing solely on the procedure may facilitate precision when not distracted by other responsibilities as reported in the literature; however, the literature findings do not support this hypothesis [27].

The demographic and clinical characteristics of the participants in both groups of our study were well-matched, with more than half of the subjects being 65 years of age or older. This is a significant consideration, as in Europe and North America, over 60% of cancer patients fall within this age bracket. The geriatric population is particularly vulnerable due to the prevalence of multimorbidity and polypharmacy [28,29].

Desaturation was the most frequent complication observed, with a higher incidence in the group receiving remifentanil, despite lower propofol usage. This finding is critical as it highlights the respiratory risks associated with opioid use. This issue was previously raised by other authors who encountered similar difficulties [25,30].

Our innovative use of the Pain Monitor device to objectively assess the sympathetic response to procedural pain represents a potential first in the field of interventional pulmonology.

The five-point Likert scale used to gauge patient satisfaction provided us with a simple yet effective means of capturing patient-reported outcomes, allowing for a straightforward comparison of results across the study [25].

In conclusion, our study provides valuable insights into the optimization of anesthesia protocols for EBUS TBNA, particularly for ASA III patients. The findings suggest that a carefully constructed, patient-specific anesthesia protocol can lead to better safety, satisfaction, and outcomes, potentially influencing future practices in interventional pulmonology. Our findings are in line with the ongoing discussion of feasible anesthetic management of more invasive diagnostic and therapeutic procedures, such as ERCP, lung biopsies, and neurointerventions like aneurysm management or CT-guided renal carcinoma ablation [31]. Several authors advocate for the integration of local and regional anesthesia with sedation [32,33,34,35]. Effective anesthetic management not only enhances patient comfort but also improves procedural accuracy and safety. By minimizing systemic analgesic requirements, we can reduce the risk of adverse events and optimize patient outcomes [36,37]. The tailored approach of using both local and regional anesthesia with sedation could represent a paradigm shift in the management of invasive diagnostic interventions, ensuring both efficacy and patient-centric care.

### Limitations

This study is subject to several limitations. Firstly, the sample size is relatively small when compared with other studies. This is a consequence of the annual number of EBUS TBNA procedures performed at our center, which is around 400, with only approximately 70 involving an anesthesiology team. Additionally, the sedation methods used in the study precluded operator-administered sedation.

Another limiting factor was the inclusion criterion, which was restricted to patients with significant comorbidities, as on average, only about 40% of patients are classified as ASA III annually. A larger study population could potentially enhance the robustness of the findings.

The assessment of patient and operator satisfaction relied on a simple five-point Likert scale. While the Polish literature describes the more comprehensive Iowa Satisfaction with Anesthesia Scale, its length and the reluctance of patients to engage with it led to its exclusion from our study.

Furthermore, the potential for an interviewer effect cannot be discounted. The same individual who conducted the study also inquired about satisfaction with anesthesia and procedural conditions, which might have influenced the high satisfaction levels reported. A more varied response might have been elicited if these questions were posed by someone unfamiliar to the patient.

Lastly, while diagnostic accuracy is a common criterion for comparing anesthesia methods in EBUS TBNA, our study did not include this measure. Previous research, such as the study by Sergio C. Conte et al., compared light sedation administered by a pulmonologist to deep sedation overseen by an anesthesiology team, assessing diagnostic accuracy, sensitivity, material quality, adverse events, and patient satisfaction, finding no significant differences between anesthesia methods. A similar conclusion was reached in a study at the University Hospital in Zurich, which found no significant differences in diagnostic accuracy or biopsy material quality when comparing general anesthesia to moderate sedation.

The reliability of anesthesia assessment in our study hinges on the consistency of the practitioner performing the procedures. Variability in operator skill can markedly influence the outcome of such evaluations. With three different operators involved in our study, we did not account for the impact of anesthesia on the diagnostic yield of the biopsies, though this remains a compelling avenue for future inquiry.

## 5. Conclusions

In conclusion, the transtracheal block, as an opioid-free anesthesia method for EBUS TBNA, stands out for ensuring patient safety, providing favorable conditions for the procedure, achieving high patient satisfaction, and minimizing sympathetic nervous system activation. This approach not only challenges traditional anesthesia paradigms but also underscores the potential for innovation in the management of patients with complex health profiles.

## Figures and Tables

**Table 1:** Comparison of demographic features between the groups.

Variable	Group I (N = 33; 58.9%)	Group II (N = 23; 41.1%)	*p*-Value
Gender (M/F)	23/10	19/4	0.27
Age (years)	66 ± 7	64 ± 11	0.47
Age ≥ 65 [N (%)]	19 (57.6)	13 (56.5)	0.94
Body weight (kg)	77.5 ± 15.7	80.6 ± 13.9	0.45
BMI (kg/m^2^)	27.1 ± 4.9	27.7 ± 4.9	0.64

**Table 2:** Comparison of indications for EBUS TBNA procedure.

Feature		Group		χ^2^ Test	Fisher’s Exact Test
		I	II		
Cancer recurrence	Yes	1 (4.3%)	1 (3.0%)	*p* = 0.638	*p* = 1.000
	No	22 (95.7%)	32 (97.0%)		
Cancer diagnosis	Yes	17 (73.9%)	22 (66.7%)	*p* = 0.776	–
	No	6 (26.1%)	11 (33.3%)		
Other diagnostics	Yes	7 (30.4%)	12 (36.4%)	*p* = 0.862	–
	No	16 (69.6%)	21 (63.6%)		

**Table 3:** Comparison of adverse events in the two groups.

Feature		Group		χ^2^ Test	Fisher’s Exact Test
		I	II		
Bradycardia	Yes	2 (8.7%)	1 (3.0%)	*p* = 0.747	*p* = 0.562
	No	21 (91.3%)	32 (97.0%)		
Desaturation	Yes	22 (95.7%)	20 (60.6%)	*p* = 0.008	*p* = 0.004
	No	1 (4.3%)	13 (39.4%)		
Apnea	Yes	3 (13.0%)	1 (3.0%)	*p* = 0.366	*p* = 0.295
	No	20 (87.0%)	32 (97.0%)		

## Data Availability

Original data is available on reasonable request.

## Supplementary Materials

The following supporting information can be downloaded at: https://www.mdpi.com/article/10.3390/jcm14061964/s1, SuppMat1_questions; SuppMat2_comorbidities.
