# Supplementary material for: Prospective Randomized Comparison of Opioid-Based Versus Non-Opioid-Based Anaesthetic Protocols for Endobronchial Ultrasound-Guided Transbronchial Needle Aspiration (EBUS-TBNA)
Source: J Clin Med. 2025 Mar 14;14(6):1964. doi: 10.3390/jcm14061964 (PMC11943042; doi:10.3390/jcm14061964)
Supplement: Supplementary file 1 [file jcm-14-01964-s001.zip › SuppMat2_comorbidities.pdf]

Comorbidity		Group		Test χ2	F Test
		I	II		
Arterial hypertension	Y	15 (65,2%)	18 (54,5%)	p=0,601	–
	N	8 (34,8%)	15 (45,5%)		
Atrial Fibrillation	Y	6 (26,1%)	7 (21,2%)	p=0,918	–
	N	17 (73,9%)	26 (78,8%)		
Myocardial infarct	Y	5 (21,7%)	8 (24,2%)	p=0,918	–
	N	18 (78,3%)	25 (75,8%)		
Ischemic Heart Disease	Y	8 (34,8%)	14 (42,4%)	p=0,766	–
	N	15 (65,2%)	19 (57,6%)		
Different heart condition	Y	5 (21,7%)	9 (27,3%)	p=0,875	–
	N	18 (78,3%)	24 (72,7%)		
COPD	Y	12 (52,2%)	9 (27,3%)	p=0,107	–
	N	11 (47,8%)	24 (72,7%)		
Asthma	Y	3 (13,0%)	2 (6,1%)	p=0,671	p=0,392
	N	20 (87,0%)	31 (93,9%)		
Emhysema	Y	2 (8,7%)	2 (6,1%)	p=0,880	p=1,000
	N	21 (91,3%)	31 (93,9%)		
Pneumoconiosis	Y	1 (4,3%)	1 (3,0%)	p=0,638	p=1,000
	N	22 (95,7%)	32 (97,0%)		
Respiratory failure	Y	3 (13%)	3 (9,1%)	p=0,975	p=0,681
	N	20 (87%)	30 (90,9%)		
Pulmonary fibrosis	Y	1 (4,3%)	2 (6,1%)	p=0,747	p=1,000
	N	22 (95,7%)	31 (93,9%)		
Different lung condition	Y	6 (26,1%)	3 (9,1%)	p=0,182	p=0,139
	N	17 (73,9%)	30 (90,9%)		
DVT	Y	0 (0,0%)	1 (3,0%)	p=0,855	p=1,000
	N	23 (100%)	32 (97,0%)		
Diabetes mellitus	Y	5 (21,7%)	2 (6,1%)	p=0,182	p=0,110
	N	18 (78,3%)	31 (93,9%)		
Stroke	Y	1 (4,3%)	4 (12,1%)	p=0,598	p=0,639
	N	22 (95,7%)	29 (87,9%)		
Different cancer	Y	2 (8,7%)	6 (18,2%)	p=0,542	p=0,449
	N	21 (91,3%)	27 (81,8%)		
