# Supplementary material for: Prospective Randomized Comparison of Opioid-Based Versus Non-Opioid-Based Anaesthetic Protocols for Endobronchial Ultrasound-Guided Transbronchial Needle Aspiration (EBUS-TBNA)
Source: J Clin Med. 2025 Mar 14;14(6):1964. doi: 10.3390/jcm14061964 (PMC11943042; doi:10.3390/jcm14061964)
Supplement: Supplementary file 1 [file jcm-14-01964-s001.zip › SuppMat1_questions.pdf]

Assessment of the procedure by the operator and by the patients:

The procedural conditions was evaluated by the operator using a five-point Likert scale. Post-procedure, the operator was asked to answer the question, **"Were the examination conditions, depending on the anesthesia, satisfactory for you?"** with the following numerical scale:

1. Definitely not
2. Rather not
3. I don't know
4. Rather yes
5. Definitely yes

Patient satisfaction with anesthesia was also evaluated using a five-point Likert scale. Approximately six hours after the conclusion of anesthesia, once fully awake, patients were asked to rate their satisfaction with the statement: "**Was the anesthesia for the EBUS TBNA examination satisfactory for you?"** The responses were quantified as follows:

1. Definitely not
2. Rather not
3. I don't know
4. Rather yes
5. Definitely yes
